# Nutrition Policy and Individual Struggle to Eat Healthily: The Question of Public Support

**DOI:** 10.3390/nu12020516

**Published:** 2020-02-18

**Authors:** Kristin Jürkenbeck, Anke Zühlsdorf, Achim Spiller

**Affiliations:** Department of Agricultural Economics and Rural Development, Marketing of Food and Agricultural Products, University of Goettingen, 37073 Göttingen, Germany; azuehls@gwdg.de (A.Z.); a.spiller@agr.uni-goettingen.de (A.S.)

**Keywords:** nutrition policy instruments, acceptability, intervention ladder, government, health status

## Abstract

The evidence for the effectiveness of nutrition policy interventions is growing. For the implementation of such interventions, social acceptability is crucial. Therefore, this study provides insight into public support for nutrition policy measures such as labelling and taxation. Further it analyses the level of acceptance in a quantitative segmentation approach. A new element to our approach is the comparison of different policy instruments, focusing on the interaction between policy acceptance and the perceived individual struggle to eat healthily. The survey was conducted in November 2017 and a total of 1035 German consumers are included in the data. The results indicate that the majority of German citizens accept nutrition policy interventions. Based on a cluster analysis, five different target groups according to the general acceptance of policy interventions and their own struggle to eat healthily are derived. The five-cluster solution reveals that both consumers who tend to eat a healthy diet as well as those who have problems with their diet support nutritional interventions. This shows that the perceived own struggle to eat healthily does not predict whether consumers accept nutrition policy interventions.

## 1. Introduction

In developed countries, the increasing number of overweight and obese people is a major public health concern [1], and dietary choices are viewed as a key factor in influencing the global burden of non-communicable diseases also known as chronic diseases [2,3]. The choice of diets that are not in keeping with dietary guidance is associated with various comorbidities such as type 2 diabetes, cardiovascular diseases and cancer [4]. In addition, to the individual consequences of an unhealthy diet, its impact on the healthcare system is politically relevant. The annual direct costs of obesity in Germany amount for approximately €29.39 billion and the indirect costs (e.g., lower productivity, long-term nursing care and pain) to an additional €33.65 billion [5]. A total of 102,000 subjects die prematurely each year because of obesity, and there is a significant excess of unemployment, long-term nursing care, pain and suffering due to obesity [5]. From a lifetime perspective, every obese man is equal to an additional burden of €166,911 and each woman of €206,526 for total healthcare costs [5]. From a more comprehensive perspective, the global economic costs caused by obesity account for 2.3% of the worlds’ gross domestic product which equals US$2 trillion [6].

To overcome this economic burden, it is necessary that overweight and obese citizens change their diet. Changing eating behaviour is a slow and long-lasting process [7] and a significant challenge for most people. In recent years, several countries have adopted policy interventions to support a healthy diet of their citizens. These interventions can range from providing information to instruments with a greater level of intervention, e.g., taxes on unhealthy foods, bans of advertisement targeted at children (for a detailed explanation of the different policy instruments see Table 1). Over the past few years, the debate on such policy instruments has gained international importance, as a result of increased efforts of the World Health Organization (WHO) [8,9,10]. For example, some European countries have introduced health-related (“fat”) taxes. In 2011, Denmark was the first country to introduce a tax on saturated fatty acids [11]. The UK established a tax on sugar-sweetened beverages in April 2018. The extra revenue is used for healthier school meals and improvements in physical education in schools. Research shows that the introduced tax in the UK was an incentive for companies to reduce the sugar content of their sugar-sweetened beverages and that it led to a reduction in sales volume of these drinks [12]. This shows that such health taxes are effective as companies reduced the sugar content in their soft drinks and, at the same time, consumers bought less of them. However, such taxes are often highly controversial. For example, the tax on saturated fatty acids in Denmark was abolished again in January 2012, after a change of government. However, the importance of a suitable policy mix for a healthy diet is increasingly recognised in the literature [9,13,14,15].

With regard to such health-related taxes or other food-related interventions, the state is reluctant in Germany as such policy interventions in the field of personal diets might be very unpopular. For example, in the 2013 election campaign, the demand for an obligatory veggie day per week (meat-free day in canteens) was perceived as a public relations disaster for the Green party, which has deterred many other politicians from proposing health-oriented interventions. Nevertheless, discussions about policy instruments, which go beyond simple health information have intensified in recent times [8]. However, some politicians and many companies have highly contested these instruments [16,17], drawing the picture of a nanny state. The results of an international comparison show great differences in terms of political intervention in the diet of consumers [18]. In respective rankings, Germany is among the countries with particularly few nutrition policy interventions, whereas many Scandinavian countries go much further [18,19].

Research about consumer acceptance of various different nutrition policy interventions within one study is scarce in Germany. Reisch et al. [20] focused on consumers’ preference for different nudges towards law and policy, most of them were designed to promote health, safety and clean energy. Their major finding was that consumers reject nudges that take their money without asking them, although the underlying reason for the nudge is attractive [20]. The review by Niebylski et al. [21] analysed healthy food subsidies and unhealthy food taxation. As a conclusion, they recommended the implementation and evaluation of healthy food subsidies and unhealthy food taxation. A tax on unhealthy food which results in a 20% price increase reduces unhealthy eating behaviour [22]. One review examined the acceptability of government interventions to change health-related behaviours [23]. The results show that the greatest public acceptability of government interventions is given for the least intrusive interventions that are often the least effective. Eykelenboom et al. [24] recommend four strategies to increase public acceptability of a sugar-sweetened beverage tax. First, communicate the differences between the general belief and the scientific evidence of such a tax. Second, use the additional revenue for health initiatives. Third, tell the purpose of the tax to the consumer in a transparent way and fourth, give political priority to the problem of policy adoption and implementation [24]. Moreover, interventions that do not affect one’s own behaviour are more accepted than interventions that target it [23].

In consumer research, many indications denote that attitudes influence behaviour, and vice versa [25,26]. In this direction, according to the current literature, the issue of whether the acceptance of health-oriented governmental interventions is related to one’s own struggle to eat healthily has not been investigated. As it is difficult for consumers to change their own eating behaviour [7] some of them might require support e.g., through policy interventions. There are some indications that one’s own eating habits may influence attitudes towards nutrition policies [27].

Thus, the main objective of this study is to explore the relationship between the general acceptance of policy interventions and the individual efforts to achieve a healthy diet. To this end, a principal component- and a cluster analysis were performed with the example of German consumers to identify different consumer segments and their attitude towards specific policy instruments.

## 2. Nutrition Policy Instruments and Public Acceptability

### 2.1. Policy Intervention Ladder and Public Evaluation

Nutrition policy interventions can be divided into three levels of engagement with increasing intervention depth: (1) Decision support, (2) decision guidance and (3) decision restrictions [28]. An overview of the different policy interventions sorted by the level of intrusiveness (bottom-up), often defined as a policy intervention ladder [29], is visualised in Table 1.

The first instruments fall into the category of supported decisions. The initial stage is characterised by the absence of state intervention in individual nutritional behaviour. The government only observes the current market situation and possible challenges. Informed choices go one step further. On this level, the government uses instruments that support more healthy food choices by consumers, such as instruments of consumer education and means of enhancing market transparency (e.g., mandatory nutrition information on food packages). Media campaigns (e.g., television, billboards and social media) about unhealthy food products such as sweets and soft drinks also belong to this category. Consumers strongly accept a ban on advertising targeted at children [23,31]. Consumers generally support interventions that provide information and knowledge about healthy eating [31]. One step further, the policy intervention ladder contains instruments for simplifying choices. Front-of-package labelling and especially the traffic-light scheme have been highly discussed instruments in recent years [32,33,34,35,36]. Traffic-light labelling scores different nutritional content in packaged foods (e.g., salt, calories, sugar and fat content)—red for unhealthy, yellow for moderate and green for healthy. Research reveals that consumers support a front-of-package label with information about saturated fat (85%), sugar (84%), total fat (83%) and sodium (78%) [37]. Hieke and Wilczynski [38] indicate that if a traffic-light label is present, consumers mainly focus on high sugar and fats when deciding between food alternatives. Other instruments in this category are health claims. Acton et al. [39] demonstrate that consumer ability to use traffic-light labelling effectively is undermined when nutrition claims are present in close proximity to the traffic-light symbol.

The next level consists of instruments that are aimed at decision guidance. Nudging describes techniques of influencing behaviour without resorting to prohibitions, rules or economic incentives. To count as a mere nudge, the intervention “must be easy and cheap to avoid” [40]. In recent years, nudging has been extensively discussed in academic research and in policy debates [41]. Placing healthy food products such as fruits and vegetables near the counter in cafeterias is a prominent example. Research indicates that consumers accept nudges such as changing the shelf location of healthier food choices [42,43].

In addition to nudging, positive and negative incentives are also aimed at changing the decision-making architecture of consumers. Cullerton et al. [44] depict that it is necessary that political will is present and that it is underpinned by public will to enable policy changes. They recommend policy makers and stakeholders work closely together to phrase policy problems and to communicate policy solutions to both, decision makers and the general public [44]. For example, encouraging healthier products through subsidies is often discussed for fruits and vegetables, whereas taxing unhealthy food should reduce consumption depending on the respective price elasticities. In Germany, only 32% of consumers agree to the introduction of a tax on unhealthy foods [31]. Current surveys indicate that acceptance may have increased in recent years [45,46]. The use of tax revenues to improve the healthcare system or the reduction of taxation on healthy food products increases public acceptance [47].

The level with the highest intervention depth includes measures for limiting individual decisions (decision restriction). In this context, state regulations for the modification of recipes for processed foods (product reformulation) or enhanced product standards such as minimum standards for school catering are to be mentioned. The mandatory fixing of maximum levels of sodium or trans fatty acids is used in many countries [48,49,50]. In 2007, the European Commission adopted a white paper on nutrition, overweight and obesity-related health issues [51] to combat unbalanced nutrition, in line with the WHO strategy. After all, the most interventional nutrition policy instruments are product bans. Consumers’ choice is restricted, for example, by banning the sale of alcoholic beverages to adolescents or soft drinks in schools.

Public support for policy instruments is important in a democracy where parties seek approval for their programs. Nutrition policy is a field with a high impact on voters. A growing number of publications suggest that a more stringent governmental nutrition policy could make sense in view of the problems of overweight and malnutrition [52,53,54]. However, governmental nutrition policy may interfere with the individual citizens’ freedom of choice. Therefore, a minimum level of acceptance by citizens is required. Reynolds et al. [15] found out that 54% of consumers accept food policy interventions and this can be increased by 3–5% when the effectiveness of the intervention is communicated to consumers. Moreover, a recent meta-analysis by Reynolds et al. [55] found out that the acceptance increases when the effectiveness of a policy is communicated and vice versa. Moreover, nutrition policy is a controversial topic with strong competition from various lobby groups, which often refer to (perceived) public opinions [56,57,58].

As explained above, only a few studies have dealt with the acceptance of individual nutrition policy instruments. However, there is little research on the social acceptance of different policy instruments. One hypothesis is that the level of intrusiveness works as an influential factor. The more intrusive the intervention is, the less it is accepted [23]. Therefore, interventions such as education are more accepted than taxation. Moreover, acceptability is driven by the perceived effectiveness of an intervention. In particular, personal and societal effectiveness and fairness play a major role in the acceptance of interventions [59]. Consumers who believe that the environment is responsible for their being overweight are more supportive [42]. Additionally, intrusive interventions are more likely to be accepted when they target commercial businesses rather than individuals. Acceptance sometimes increases after the introduction of policy interventions [23].

A sub target of the study is to analyse the acceptance of different policy interventions in comparison, whereby the following instruments are examined: (1) colour-based nutritional labelling, (2) restrictions in food marketing aimed at children and (3) limits of the maximum amount of sugar, fat and salt in foods. Moreover, various types of consumption taxes or levies were considered: (4) A revenue-neutral soft drink tax (revenue-neutral depicts that the tax for soft drinks is increased and the tax for fruits and vegetables decreased, the amount of tax paid should be the same as before the tax introduction), (5) an animal welfare levy, (6) a tax on foods with a very high sugar, fat, or salt content without compensation (non-revenue-neutral), (7) a revenue-neutral tax on foods with a very high sugar, fat, or salt content, where the additional revenue would be used to reduce the price of healthy foods. It is revenue-neutral because consumers who follow a balanced diet do not have to expect higher costs. Lastly, (8) for an earmarked tax, the revenues of which are reserved for improved health prevention. These instruments were included in the study because they are extensively discussed in public debates in Germany. In addition, the general attitudes towards nutrition policy were analysed.

### 2.2. Vulnerability and Governmental Support

Research results confirm a clear differentiation of nutritional behaviour, from very health-oriented consumers to carefree eaters [60]. Many studies have established a strong correlation between social status and eating habits [61,62]. People with lower education levels and lower incomes are disproportionately characterised by an unhealthier diet and significantly higher obesity rates. However, hardly any studies have investigated the relationship between individual eating behaviour and the acceptance of policy interventions, and if so, the results are controversial.

Some related studies in the literature indicate that the influence of individual nutritional behaviour and personal health status on the acceptability of nutrition policy interventions can be contradictory. Consumers who are aware of their health status are more likely to endorse behavioural measures, whereas consumers with problematic eating habits without knowledge tend to reject behavioural measures [31]. By contrast, consumers with a high body mass index (showing that they have dietary problems) have a positive attitude towards advertising restrictions and are willing to pay higher taxes for healthier school lunches [27]. However, most Americans do not want to reduce the amount of fast food provided in schools [27]. Diepeveen et al. [23] have suggested that people with a high body mass index are less supportive of food labels addressing obesity. Oliver et al. [27] state that consumers who physically exercise tend to support policies that address obesity.

Further, past research has assessed the influence of sociodemographic characteristics on the acceptance of nutrition policy interventions. The influence of income on the acceptance of nutrition policy interventions is unclear. Diepeveen et al. [23] revealed that consumers with a lower income are more supportive, whereas other studies found that they are less supportive than consumers with a higher income. Additionally, female and older consumers are apparently more supportive of policy interventions [23].

## 3. Materials and Methods

### 3.1. Data Collection and Survey Design

Before carrying out the empirical survey in November 2017, a pre-test was conducted including marketing experts and consumers. After the pre-test some questions and related statements were modified to ensure common understanding. The questionnaire was mounted within “Questback EFS Fall 2019” and a professional online access panel provider (respondi) was responsible for data collection. Thus, the panel provider distributed the URL of the questionnaire to their clients who could then in turn choose to participate. In order to ensure representativeness and to preserve the possibilities of multivariate data analysis, the literature recommends sample sizes between 200 and 1200 respondents [63]. Quotas were set for gender, age, education and region to obtain a sample that is representative of the German population with respect to these characteristics. The respondents had to state in which federal state of Germany they live. The federal states were classified into regions as north, south, east and west. Strict data quality checks were performed before analysing the data to ensure reliability. First, 23 respondents with less than one third of the average response time, and second, 19 straight-liners (respondents who answered in a stereotypical manner) were excluded. A total of 1035 respondents remained in the sample for analysis.

At the beginning of the online questionnaire, participants had to answer several sociodemographic questions such as gender, age, and income. Afterwards, the participants had to evaluate statements about their general opinion regarding governmental interventions to support healthy eating behaviour, and statements about their own eating behaviour (see Appendix A), using a five-point Likert scale ranging from +2 (“I totally agree”) to −2 (“I do not agree at all”). The selection of statements about the different policy interventions was based on prior research, and the statements were modified to fit the research objective and the German sample [64,65]. The statements about one’s own struggle to eat healthily were developed by the authors. These statements build the foundation for the principal component analysis. Afterwards, various statements about specific policy interventions had to be evaluated on the same five-point Likert scale.

### 3.2. Data Analysis

Data analysis was conducted using IBM SPSS 24 statistical software. At the descriptive level the top box values (percentage frequencies of +2 and +1 as agreement, 0 as neither nor and −1 and −2 as rejection) and the 95% confidence intervals of the statements for the general opinion about nutrition policy instruments as well as the statements of the specific policy instruments were calculated (Appendix A, Table A1 and Table A2). The exploratory data analysis consisted of two steps. First, principal component analysis (PCA) with varimax rotation of the general statements about policy interventions was performed to reduce the complexity of the data. The Kaiser–Meyer–Olkin (KMO) criterion provides information about sampling adequacy. During the first PCA, two factors emerged. However, for the second factor about the individual eating behaviour the Cronbach’s alpha value remained below the recommended threshold [66] (Appendix A, Table A3). Therefore, two of the three statements about the individual eating behaviour were excluded. 

A new PCA was conducted. After the renewed PCA, the second factor consisted of only one item. Normally, a construct of factor analysis is represented by several items to cover as many influencing factors as possible and thus increase the extracted variance [67]. In case of a single item factor, there should be a strong assumption that the item represents the factor. The authors assume that the second factor (“perceived struggle to eat healthily”) is well represented by one item (“it’s hard for me to eat healthily”), since both the item and the factor deal with the perceived problem of eating healthily. Internal consistency for the first factor was tested with Cronbach’s alpha.

In a second step, cluster analysis was performed to obtain homogeneous groups based on the factor values from the PCA. Outliers were initially identified using single linkage clustering. To obtain the number of classes, Ward algorithms including the elbow criteria and a dendrogram were used to identify the best solution. Hence, a five-cluster solution was selected, and the cluster centroids were utilised as starting points for the following k-means clustering method. Group membership was classified by k-means. Discriminant analysis was performed to validate the accuracy of the classification.

Moreover, the statements about the specific policy interventions were used as cluster-descriptive variables to compare them. Thus, mean scores were calculated for the entire sample and for each cluster. An analysis of variance (ANOVA), with the respective post hoc tests, was conducted as well. As post hoc tests, Tukey or Games Howell was used depending on whether Levene’s test was significant. For the sociodemographic variables, chi-square test in cross tabulation including z-test was conducted.

## 4. Results

### 4.1. Sample Description

The sample consisted of 1035 German citizens. The quota setting for gender, age, education and region worked in a manner that effectively mimics these characteristics of the German population (Table 2).

On average 63.4% of respondents agree with the general statements about nutrition policy interventions by the government (Appendix A, Table A1). However, the query of specific examples of policy instruments shows lower acceptance levels. The average acceptance rate of all instruments amounts to 50.9% (Appendix A, Table A2). Accordingly, the rejection of nutrition policy instruments increases when specific examples are given (general statements: 11.2% and specific nutrition policy instruments: 29.6%) (Appendix A, Table A1 and Table A2).

### 4.2. Results of the Principal Component Analysis

The PCA revealed two factors (Table 3). The KMO of 0.847 is acceptable [69], and the Cronbach’s alpha value denotes that the items are reliably measured within one factor [66]. Factor 1 that is named “general support of nutrition policy interventions” has a Cronbach’s alpha value of 0.860. The second factor is called “perceived struggle to eat healthily”.

The values of the extracted factors were used in the subsequent cluster analysis as cluster-building variables.

### 4.3. Results of the Cluster Analysis

The cluster analysis based on the two factors described above resulted in five consumer segments. The first group consisted of 17.3%, the second of 12.3%, the third of 25.5%, the fourth of 12.1% and the fifth of 32.8% of the sample. The discriminant function indicated that 97.3% of the cases were classified correctly. Table 4 visualises the mean values of the items included in the two factors. Compared to the factor means, the mean values of the items are better for interpretation, as the direction of agreement or disagreement can be observed directly.

Cluster 1 demonstrates the strongest support of nutrition policy interventions. This cluster struggles to eat healthily. Compared to the other clusters, cluster 1 has the greatest struggle to eat healthily.

Cluster 3 slightly supports nutrition policy interventions. Its support is weaker than from clusters 1 and 5. Cluster 3 marginally struggles to eat healthily.

Cluster 5 supports nutrition policy interventions, the second strongest after cluster 1. However, cluster 5 does not struggle to eat healthily compared to cluster 1.

Two other smaller groups (namely, 2 and 4, make up a total of 24% of the respondents) are slightly opposed to nutrition policy interventions. On the one hand, cluster 2 includes respondents who struggle to eat healthily. On the other hand, cluster 4 comprises respondents who do not struggle to eat healthily.

Cluster 2 rejects nutrition policy interventions. The rejection is the second strongest among all clusters. The rejection is somewhat lower than that of cluster 4. Similar to cluster 1, cluster 2 struggles to eat a healthy diet. However, cluster 2’s struggle to eat healthily is somewhat smaller than that of cluster 1.

Cluster 4 rejects nutrition policy interventions. Its rejection is the strongest in comparison to the other four clusters. Furthermore, cluster 4 has no perceived struggle to eat healthily. Clusters 4 and 5 identically do not struggle to eat healthily.

Based on the above differences, the clusters are labelled as follows: “Help-seeking advocates” (1), “health-unconscious rejecters” (2), “differentiating supporters” (3), “health-conscious rejecters” (4) and “health-conscious advocates” (5). Furthermore, the authors analysed if the five clusters differ in their acceptance of specific nutrition policy instruments by conducting an ANOVA (Table 5). The specific nutrition policy instruments are used as cluster-descriptive variables.

The help-seeking advocates (1) have the highest acceptance for the coloured traffic-light marking on the front of food packaging, followed by setting product limits and the lowest acceptance for a tax on foods with a high content of sugar, fat or salt without compensation. Notably, help-seeking advocates express higher support for setting product limits than imposing a ban on marketing to children.

The health-unconscious rejecters (2) only support the coloured traffic-light marking on the front of food packaging. They reject all other specific nutrition policy instruments. They demonstrate the weakest rejection for setting product limits. At the same time, this intervention is the most intrusive in this study. Furthermore, the health-unconscious rejecters (2) refuse nutrition policy instruments that include a tax with compensation to roughly the same extent. However, they most strongly reject a tax without compensation.

The differentiating supporters (3) reveal a high approval for the coloured traffic-light marking on the front of food packaging. They show a lower acceptance for setting product limits. The evaluation of the other specific nutrition instruments is indifferent (i.e., they neither support nor reject these nutrition instruments). The differentiating supporters (3) only clearly reject the tax without compensation.

The health-conscious rejecters (4) only support the coloured traffic-light marking on the front of food packaging. They most strongly reject a tax that is used to improve healthcare. Somewhat less strongly, they refuse a tax without compensation. Their rejection of the remaining specific policy instruments is moderately strong.

The health-conscious advocates (5) most strongly support the coloured traffic-light marking on the front of food packaging. They express the second highest support for setting product limits. Their support for other specific policy instruments is moderate. Their lowest level of support is for a tax without compensation.

Overall, all clusters show the highest acceptance values for a coloured traffic-light marking on the front of packaging. Four out of five clusters demonstrate the lowest acceptance or highest rejection of a tax on foods with a high content of sugar, fat or salt without compensation. Additionally, this is the only instrument that the entire sample rejects.

Descriptive statistics indicate that the clusters significantly differ in some sociodemographic characteristics (Table 6).

The cluster of differentiating supporters (3) consists of the youngest consumers, with an average age of 45.1 years, whereas the cluster of health-conscious advocates (5) represents the oldest consumers, with an average age of 53.1 years. In terms of education, health-conscious rejecters (4) on average have the highest educational background. In this context, the cluster of health-conscious rejecters (4) has the highest amount of consumers with an income above €4500. At the same time, this cluster includes the lowest number of individuals with a salary below €1300.

## 5. Discussion

The study results show that the majority of the German population is open to nutritional policy interventions by the state. Of the German consumers, 63.4% support nutrition policy in general, 11.2% have a negative attitude and 25.4% are indifferent (Appendix A Table A1). These findings are in line with the literature [42,70,71]. The assessment of concrete instruments gives a more complex picture and shows approval ratings of between 34.2% and 78.7% depending on the instrument (50.9% on the average of all instruments, Appendix A Table A2). This might be a consequence of the fact that the personal impact of a policy measure influences the evaluation of the instrument.

In the present study one’s perceived own struggle for a healthy diet has been analysed with regard to the hypothesis of a connection between personal vulnerability and the acceptance of nutritional policy measures. Overall the results depict that the extent of support for food policy instruments is not linearly related to one’s perceived own struggle for a healthy diet. The cluster analysis identifies both consumer groups who are themselves struggling for a healthy diet as supporter (or rejecter) of nutrition policy interventions, as well as consumers who do not have any difficulties themselves. In fact, only a minority of the population (size of cluster 2 and 4) clearly rejects nutrition policy interventions, whereas most of the population (size of cluster 1, 3 and 5) is more or less open-minded.

**Figure 1:**
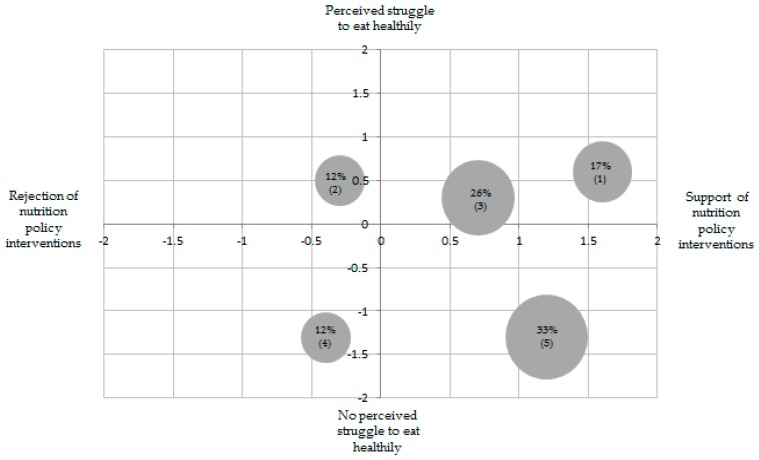
Cluster matrix (mean values). Notes: Numbers are the index values for each cluster, the mean values are added up of each variable and divided by the number of interventions (see Table 4); scale from: −2 = “I do not agree at all” to +2 = “I totally agree”; from left to right: 12% = health-conscious rejecters (4), 12% = health-unconscious rejecter (2), 26% = differentiating supporters (3), 33% = health-conscious advocates (5); 17% = help-seeking advocates (1).

The results of the present study, which illustrates that consumers agree to nutrition policy instruments unrelated of their own efforts for a healthy diet, partly differ from previous literature. A past study has revealed that the acceptance of nutrition policy measures is higher among people who eat unhealthily than among individuals who eat healthily [31]. However, the current study focuses on the personal struggle for a healthy diet, not on the individual eating behaviour as an outcome of the personal efforts. Figure 1 visualises the non-linear results of the present study in a two-dimensional matrix.

The idea that nutrition policy interventions are not solely aimed at one target group is important to recognise. According to the present study results, German citizens may be divided into five target audiences with different policy intervention preferences. Thus, target group specific political communication strategies (i.e., the different segments should be targeted separately with individual communication strategies) are advised [72]. For example, a communication campaign targeting health-unconscious rejecters (2) should explain how policy interventions might help them to follow a healthier diet. They could take the given health information into account which might help them to shift their food purchase towards a healthier choice.

Overall, our results reveal a relatively positive attitude towards the acceptance of nutrition policy instruments in Germany. As expected, information tools are more readily accepted than taxes or bans [23]. All five clusters support traffic-light labelling on the front of packaging. This finding is consistent with earlier research [37]. It leads to the following clear conclusion that politicians can be confident to introduce e.g., the traffic-light label as a mandatory label on food packaging.

In contrast to our results, Effertz [31] argued that both, taxes with and without compensation are more likely to be rejected by the population. The current results reveal that consumers accept food taxes on unhealthy products when combined with a tax reduction for healthy products. Thus, food taxes including compensation are favourable. A clear communication on which products would be affected by a higher price due to a tax increase and which product prices would in turn decrease due to a tax reduction is recommended. Only if the consumers have this knowledge they might be able to adapt their behaviour accordingly and to understand the mechanism behind the tax introduction.

Other studies revealed that a ban on marketing targeted at children is the instrument that the population is most likely to support [23,31]. On the one hand, this finding is consistent with Effertz [31] as about 2/3 of his and our sample accept a ban on marketing addressed to children. On the other hand, Effertz [31] result is inconsistent with our results, as in the present study the traffic-light labelling exhibits a higher support than the ban targeted at children. Overall, a ban on marketing to children depicts high acceptance rates. Since it has been shown in several studies that a ban is supported by a large part of the population [23,31]. Politicians could take a closer look at this measure and use marketing restrictions to children as a tool to protect children in particular from a too strong influence through marketing. Further considerations in this regard could also include social media marketing.

The complex results achieved here could be explained from an economic, sociological or psychological perspective. Neo-classical economic theory [73] maintains that consumers behave according to their own preferences and reject any restrictions of their own choices [74] which is not the case in the present study. Especially cluster 1 seeks governmental support. From a neoclassical viewpoint, consumers also have a desire to maximise their utilities (own preferences). Therefore, one would expect consumers who follow these principles to evaluate the interventions according to whether or not they benefit financially from them. A fat tax makes unhealthy eating behaviour more expensive and healthy eating behaviour cheaper. According to this hypothesis, consumers with an unhealthy behaviour (i.e., cluster 1, 2, 3) should be against health taxes whereas consumers with a healthy behaviour should support them. In fact, health-unconscious rejecters (2) and health-conscious advocates (5) behave exactly as expected from neo-classical economic theory, while the health-conscious rejecters (4), differentiating supporters (3), and help-seeking advocates (1) do not behave according to this economic hypothesis. Without changing their current behaviour, health-unconscious rejecters (2) struggle to eat healthily, which is why a tax on unhealthy food would lead to higher expenses for them while the health-conscious advocates (5) would benefit financially as healthy food gets cheaper. Help-seeking advocates (1) highly accept interventions but struggle to follow a healthy diet. As members of this cluster follow an unhealthy diet, a rejection of interventions would have been expected because a health tax would affect this cluster financially—at least if they do not fundamentally change their eating behaviour.

One option to convince health-unconscious rejecters (2) of policy interventions is to communicate personal benefits of healthy behaviour. For example, if health-unconscious rejecters (2) will lose weight, they will save money in the future due to lower healthcare costs caused by their current overweight. Research shows that political interventions can change consumer behaviour [75,76]. Sharma et al. [77] estimated through panel data that a 20% price increase of sugar-sweetened beverages would lead to a reduction of consumption which results in a lower calorie intake of 10,000 kj per year. This is equivalent to 0.93 kg body weight at an average cost per household of €17. Furthermore, in countries such as Germany where health insurance is compulsorily deducted from income [78], economic costs are another factor. Consumers who follow a healthy diet should be interested in the fact that the remaining consumers eat healthily as well, so that they do not have to co-finance their healthcare costs. This aspect might additionally explain the high acceptance of political interventions by the health-conscious advocates (5). However, the explanations from an economical viewpoint depict that not all clusters can be explained accordingly and that other perspectives have to be taken into account.

A sociological perspective may also contribute to the explanation of the results. Thus, a slim appearance can be interpreted as a characteristic of social differentiation [61]. A healthy, slim appearance tends to be associated with social success, whereas weight problems are perceived as a lower-class problem [79]. Previous research indicated that higher-quality diets including regular fruit and vegetable intake are more often consumed by better-educated people [61,62,80]. According to the sociological construct of conspicuous consumption [81], upper-class citizens are interested in keeping such differences (e.g., the image of poor and fat). Therefore, they might be rather reluctant in advocating nutrition interventions. The health-conscious rejecters (4) and the health-conscious advocates (5) are the best-educated consumer segments (see Table 6). Thereby, it is expected that both reject policy interventions to keep appearance differences (slim versus fat) of social classes. However, the results show that only the health-conscious rejecters (4) judge policy interventions negatively and behave as expected from a conspicuous consumption perspective. Against the background that studies reveal different class patterns in the food consumption of healthy and unhealthy products [61], the persistent tendency of class-specific eating habits [79] may prevent the acceptance of food policy interventions at least for this cluster.

From a psychological viewpoint, reactance effects [82] can play a crucial role. Psychological reactance is a complex defensive reaction that can be understood as resistance to external or internal restrictions. Reactance is usually triggered by psychological pressure or the restriction of freedom [82]. Reactance as an acceptance barrier would result in the fact that the more health-conscious a cluster is, the higher the acceptance of policy interventions. In this context, the help-seeking advocates (1) do not behave as expected as they highly accept policy interventions, although they struggle to follow a healthy diet and thereby would experience restrictions of their own behaviour. In contrast, the attitude of cluster 2 could perhaps be explained by reactance.

Another explanation from a psychological viewpoint could be that the individual motivation [83] to change one’s habitual behaviour to follow a healthier diet is important. Therefore, the help-seeking advocates (1) may lack motivation of their own and need motivation and/or restrictions by a third party to be able to change their eating behaviour. One possible reason why consumers need support is self-commitment [84]. One reason for this could be that consumers are unable to resist the temptations of an obesogenic environment and realise their own weaknesses.

Moreover, the effect of short-term versus long-term preferences could help to explain consumers’ attitude towards nutrition policy instruments. Consumers who are aware of their short-term weaknesses should strive for self-commitment by supporting nutrition policy interventions, i.e., a healthy nutrition environment, and vice versa. The health-conscious rejecters (4) and health-unconscious rejecters (2) may exhibit a rejection due to the underestimation of the long-term consequences of an unbalanced diet. When these segments would be more aware of the future benefits (e.g., lower risk for developing type 2 diabetes, cardiovascular diseases or cancer), their members might rethink their behaviour and support policy interventions. One way to increase acceptability would be to target the health-unconscious rejecters (2) by a communication strategy which explains them that in the short-term the tax might increase their food expenditure but in the long run they could benefit. If they change their diet, they will not experience higher food expenses and they will safe health-care expenditure due to no occurrence of comorbidities related to obesity. Thus, in the long-term, they will benefit both, from a financial and health-related point of view. Communication between politicians and consumers is crucial. Only if consumers understand that these health taxes are introduced for their own benefit and not for the state to raise money, there is a chance for a change in their opinion about nutrition policy interventions.

One limitation of this research is that nutritional problems were only recorded on the basis of self-declarations about the own struggle to follow a healthy diet, the nutritional status was not measured. Similarly, the stated preferences may be biased by social desirability effects. A third limitation is that the second factor consists of only one item; therefore, the perceived own struggle to eat healthily needs to be better understood in a further study. One option would be to measure the actual health status, e.g., the body mass index.

## 6. Conclusions

Overall, the results show that about 63.4% of the German population is in favour of state policy interventions. Politicians should therefore carefully analyse the current situation and develop appropriate instruments to support citizens to follow a healthy diet. This is important because the economic burden of obesity is increasing for the whole society [5,6].

The present study is the first empirical research that takes consumers’ struggle to eat healthily into account when analysing the acceptability of different policy interventions. In this study, there are two clusters that eat healthily but their acceptance of nutritional policy instruments is contradictory. The same applies to the three clusters with rather unhealthy eating behaviour. First attempts to interpret the results are presented. However, further research is needed based on actual eating behaviour and more socio-psychological items.

## Figures and Tables

**Table 1:** Policy interventions for changing behaviour.

**Decision Restriction**	Limited selection through product bans	Changing behaviour through product bans, e.g., prohibition of alcoholic beverages, prohibition of selling soft drinks at school, prohibition of certain portion sizes
	Limited selection through product reformulation and governmental product standards	Implementing behavioural change through enforced product reformulation, e.g., maximum content of certain ingredients (e.g., salt, sugar, fat), obligatory nutrition standards in day care, schools, in public catering and health facilities
**Decision Guidance**	Guided selection through negative incentives	Initiating changes in behaviour through negative incentives, e.g., taxes and fees
	Guided selection through positive incentives	Initiating changes in behaviour through positive incentives, e.g., subsidies and bonus programs
	Guided selection through nudging	Initiating changes in behaviour by changing the default setting, e.g., preferred placement of healthy products in public catering, attractive product design
**Decision Support**	Simplified choice	Facilitate behavioural change: Nutritional counselling, feedback systems/apps, interpretative labels such as the traffic-light system, warning labels, health claims
	Informed choice	Improve decision: Consumer education and information, increasing market transparency, mandatory nutrition information, advertising restrictions, and advertising bans
	Governmental unregulated food choice	Observing the situation through governmental monitoring

Source: Author’s presentation based on [28,29,30].

**Table 2:** Sample description.

Variable	Characteristics	Sample (%)	German Population * (%)
Gender	Male	49.4	49.1
	Female	50.6	50.9
Age	16–29	15.7	18.9
	30–49	32.2	30.7
	50+	52.1	50.4
Education	No graduation (yet)	3.9	3.7
	Certificate of Secondary Education	34.0	33.0
	General Certificate of Secondary Education	30.8	29.4
	General qualification for university entrance	14.0	13.2
	University degree	17.4	16.3
Region	North	16.3	16.1
	West	36.1	35.3
	East	20.8	19.8
	South	26.9	28.8
Attitude towards nutrition policy interventions	General agreement on nutrition policy	63.4	-
	Agreement on specific policy instruments	50.9	-

Source: * Federal statistical office [68], Attitude values are top boxes and index of all items, for more details of the attitudes towards nutrition policy interventions see Appendix A, Table A1 and Table A2.

**Table 3:** Results of principal component analysis and Cronbach’s alpha.

**Factors**	**Statements**	**Factor Loading**	**Mean/SD**
1. General support of nutrition policy interventions (Cronbach’s alpha: 0.860)			
	I would be happy if the government provided healthier food	0.875	0.72/1.05
	Given the high cost of healthcare, the state must help citizens to eat healthily	0.841	0.73/1.03
	The state should ensure that we are not only influenced by the marketing of the food industry	0.792	0.78/1.01
	The Government should stay out of citizens’ diet	−0.774	0.32/1.16
	I think it would be good to label healthy food more clearly	0.726	1.16/0.84
2. Perceived struggle to eat healthily			
	It’s hard for me to eat healthily	0.995	−0.33/1.06

Notes: 70.70% of total variance explained; Kaiser–Meyer–Olkin (KMO) = 0.847; scale from: +2 = “I totally agree” to −2 = “I do not agree at all”.

**Table 4:** Means of the cluster-building variables.

Items	Cluster 1	Cluster 2	Cluster 3	Cluster 4	Cluster 5	Sample
*n* (%)	174 (17.3)	123 (12.3)	256 (25.5)	121 (12.1)	329 (32.8)	1003 (100)
1. General support of nutrition policy interventions						
Factor means	1.04	−1.29	−0.09	−1.39	0.51	0.00
Index of all items	1.60	−0.30	0.70	−0.40	1.20	0.75
I would be happy if the government provided healthier food. ***	1.77 ^b^ (0.46)	−0.41 ^a^ (0.78)	0.67 ^c^ (0.58)	−0.57 ^a^ (0.90)	1.14 ^d^ (0.73)	0.73 (1.04)
Given the high cost of healthcare, the state must help citizens to eat healthily. ***	1.61 ^b^ (0.64)	−0.33 ^a^ (0.86)	0.66 ^c^ (0.60)	−0.51 ^a^ (0.90)	1.17 ^d^ (0.71)	0.73 (1.02)
The Government should stay out of citizens’ diet. ***	−1.15 ^b^ (0.84)	0.79 ^a^ (0.96)	−0.21 ^c^ (0.82)	0.88 ^a^ (1.00)	−0.82 ^d^ (0.92)	−0.32 (1.15)
The state should ensure that we are not only influenced by the marketing of the food industry. ***	1.64 ^b^ (0.54)	−0.19 ^a^ (0.83)	0.75 ^c^ (0.66)	−0.39 ^a^ (0.95)	1.15 ^d^ (0.76)	0.78 (1.00)
I think it would be good to label healthy food more clearly. ***	1.81 ^b^ (0.42)	0.26 ^a^ (0.76)	1.07 ^c^ (0.59)	0.41 ^a^ (1.01)	1.48 ^d^ (0.58)	1.15 (0.83)
2. Perceived struggle to eat healthily						
Factor means	0.92	0.76	0.62	−0.91	−0.92	0.00
It’s hard for me to eat healthily. ***	0.65 ^a^ (0.77)	0.47 ^a,b^ (0.65)	0.33 ^b^ (0.56)	−1.30 ^c^ (0.46)	−1.31 ^c^ (0.46)	−0.33 (1.06)

Notes: Question before respondents had to rate the statements: “Should the government promote healthy eating more strongly? Please answer”; *n* = number of respondents; significance level of the F-test: *** *p* < 0.0001, the index for each cluster includes the sum of the mean values of all general policy intervention statements divided by the number of items, means (standard deviation); different letters a, b, c, d indicate a significant (*p* < 0.05) difference between groups according to Games–Howell 0.05, scale from: +2 = “I totally agree” to −2 = “I do not agree at all”. As Table 4 illustrates, three clusters (namely 1, 3, and 5, which make up a total of 76% of respondents) have a positive view of nutrition policy interventions. As an example, the three clusters differ in terms of the extent to which they struggle to eat healthily.

**Table 5:** Evaluation of specific policy instruments by the five clusters.

Nutrition Policy Instruments	Help-Seeking Advocates (1)	Health-Unconscious Rejecters (2)	Differentiating Supporters (3)	Health-Conscious Rejecters (4)	Health-Conscious Advocates (5)	Sample
Index of all items	0.90	−0.52	0.19	−0.46	0.68	0.32
The state should set product limits for sugar, fat and salt which should not be exceeded. ***	1.24 ^a^ (1.02)	−0.44 ^b^ (1.00)	0.44 ^c^ (0.93)	−0.51 ^b^ (1.09)	0.88 ^d^ (1.06)	0.50 (1.19)
Should the state increase the taxes/fees on foods with a very high sugar, fat or salt content? ***	0.23 ^a^ (1.37)	−0.95 ^b^ (1.07)	−0.38 ^b^ (1.14)	−0.93 ^b^ (1.04)	0.21 ^a^ (1.38)	−0.21 (1.33)
Should the state increase the price of meat through a tax or levy and use the money to improve animal welfare? ***	0.44 ^a,c^ (1.29)	−0.60 ^b^ (1.19)	0.17 ^a^ (1.14)	−0.47 ^b^ (1.23)	0.46 ^c^ (1.20)	0.13 (1.27)
Should the state increase taxes/fees on foods with a very high sugar, fat, or salt content and use the money to improve healthcare? ***	0.82 ^a^ (1.28)	−0.77 ^b^ (1.16)	−0.13 ^c^ (1.30)	−1.08 ^b^ (0.94)	0.46 ^a^ (1.32)	0.03 (1.39)
Should the state increase taxes/fees on soft drinks (such as cola and orange soda) and reduce that on fruit and vegetables? ***	0.90 ^a^ (1.30)	−0.61 ^b^ (1.32)	0.16 ^c^ (1.23)	−0.37 ^b^ (1.36)	0.78 ^a^ (1.21)	0.33 (1.37)
Should the state increase taxes/fees on food with a very high sugar, fat or salt content and the same time reduce taxes on healthy food (overall, the tax would remain the same)? ***	0.96 ^a^ (1.28)	−0.61 ^b^ (1.26)	0.12 ^cd^ (1.06)	−0.47 ^b,c^ (1.22)	0.52 ^a,d^ (1.30	0.24 (1.32)
I find a coloured “traffic-light” marking on the front helpful. ***	1.56 ^a^ (0.79)	0.51 ^b^ (1.05)	1.13 ^c^ (0.86)	0.67 ^b^ (1.13)	1.37 ^a^ (0.81)	1.15 (0.96)
Should the state intervene more in the area of marketing to children and ban marketing of certain unhealthy foods to children (e.g., advertising sweets on children’s television, on websites, and in online games for children, on posters and products, for example through comic figures)? ***	1.06 ^a^ (1.29)	−0.71 ^b^ (1.60)	0.02 ^c^ (1.49)	−0.49 ^b,c^ (1.63)	0.79 ^a^ (1.38)	0.36 (1.57)

Notes: *n* = number of respondents; significance level of the F-test: *** *p* < 0.0001, means (standard deviation); the index for each cluster includes the sum of the mean values of all specific policy items divided by the number of items, different letters a, b, c, d indicate a significant difference (*p* < 0.05) between clusters according to post hoc test Tukey or Games–Howell depending on whether Levene’s test was significant, scale from: +2 = “I totally agree” to −2 = “I do not agree at all”; Likert scale of statement about marketing of children was transformed from an originally 10-point scale to a 5-point scale, scale from 1 and 2 = +2 “Children’s marketing for food with a lot of fat/sugar/salt should be banned in any case” to 9 and 10 = −2 “Children’s marketing for food with a lot of fat/sugar/salt should not be banned in any way”, n of the statements about a soft drink tax, meat tax and tax using for healthcare are from a split-sample design (3 splits), The different types of grey signify different steps explained in the policy interventions for changing behaviour (Table 1).

**Table 6:** Comparison of the sociodemographics of the clusters.

Socio-Demographics	Help-Seeking Advocates (1)	Health-Unconscious Rejecters (2)	Differentiating Supporters (3)	Health-Conscious Rejecters (4)	Health-Conscious Advocates (5)	Sample
Gender (female in %)	46.6 ^a^	43.9 ^a^	53.5 ^a^	46.3 ^a^	54.1 ^a^	50.4
Age (years)	47.6 ^a,b^	47.2 ^a,b^	45.1 ^b^	51.4 ^a,c^	53.1 ^c^	49.2
Education (in %)						
No graduation (yet)	4.0 ^a,b^	1.6 ^b^	6.3 ^a^	3.3 ^a,b^	3.3 ^a,b^	4.0
Certificate of Secondary Education	39.7 ^a,b^	48.0 ^b^	32.8 ^a,c^	27.3 ^c^	29.2 ^c^	34.0
General Certificate of Secondary Education	32.2 ^a^	30.1 ^a^	31.6 ^a^	27.3 ^a^	30.7 ^a^	30.7
General qualification for university entrance	9.8 ^a^	10.6 ^a,b^	14.5 ^a,b^	19.0 ^b^	15.8 ^a,b^	14.2
University degree	14.4 ^a,b,c^	9.8 ^c^	14.8 ^b,c^	23.1 ^a^	21.0 ^a,b^	17.1
Income in € (in %)						
below 1300	30.5 ^a^	29.3 ^a^	23.4 ^a,b^	15.8 ^b^	24.5 ^a^	24.8
1300–2599	37.4 ^a,b^	41.5 ^a,b^	43.3 ^b^	32.5 ^a^	33.7 ^a^	37.6
2600–4499	25.9 ^a,b^	21.1 ^b^	25.4 ^a,b^	40.0 ^c^	31.0 ^a,c^	28.5
above 4500	6.3 ^a^	8.1 ^a^	7.9 ^a^	11.7 ^a^	10.7 ^a^	9.0

Note: different letters (a, b, c) indicate a significant difference (*p* < 0.05) between clusters according to chi-square test in cross-tabulation and z-test.

## Appendix A

**Table A1:** Top box values and confidence intervals of cluster building variables.

	++/+ (CI 95%)	0 (CI 95%)	−/−− (CI 95%)
Index of all items	63.4	25.4	11.2
I would be happy if the government provided healthier food.	61.7 (56.0–67.9)	26.8 (24.4–29.6)	11.5 (8.4–13.9)
Given the high cost of healthcare, the state must help citizens to eat healthily.	62.6 (57.1–68.7)	27.0 (24.0–29.5)	10.4 (7.8–13.2)
The Government should stay out of citizens’ diet	20.8 (16.8–24.7)	32.8 (29.6–35.6)	46.4 (42.0–51.9)
The state should ensure that we are not only influenced by the marketing of the food industry.	65.0 (59.2–71.1)	25.2 (22.8–28.1)	9.8 (7.2–12.4)
I think it would be good to label healthy food more clearly.	81.3 (75.2–87.5)	15.2 (13.0–17.6)	3.5 (2.1–5.3)

Note: scale from +2 (I totally agree) to −2 (I totally disagree), numbers are percentages of the top box values: +2 and +1 = ++/+, 0 = 0, −1 and −2 = −/−−; Confidence Interval (CI) of the lower and upper 95%.

**Table A2:** Top box values and confidence intervals of cluster descriptive variables.

Nutrition Policy Instruments	++/+ (CI 95%)	0 (CI 95%)	−/−− (CI 95%)
Index of all items	50.9	19.5	29.6
The state should set product limits for sugar, fat, and salt which should not be exceeded.	53.7 (48.1–59.0)	26.0 (23.4–28.6)	20.3 (16.7–24.2)
Should the state increase the taxes/fees on foods with a very high sugar, fat, or salt content?	34.2 (26.1–42.8)	19.8 (15.6–24.3)	46.1 (37.5–55.1)
Should the state increase the price of meat through a tax or levy and use the money to improve animal welfare?	42.0 (37.0–47.2)	26.0 (23.3–28.6)	32.0 (27.4–36.5)
Should the state increase taxes/fees on foods with a very high sugar, fat, or salt content and use the money to improve healthcare?	43.4 (34.9–52.2)	17.9 (13.8–22.0)	38.7 (30.5–46.9)
Should the state increase taxes/fees on soft drinks (such as cola and orange soda) and reduce that on fruit and vegetables?	53.6 (48.3–58.9)	15.4 (13.4–17.7)	31.0 (26.6–35.3)
Should the state increase taxes/fees on food with a very high sugar, fat, or salt content and therefore reduce taxes on healthy food (overall, the tax is to be reduced to remain the same)?	48.3 (39.8–57.7)	21.0 (17.0–25.3)	30.7 (23.6–38.3)
I find a coloured “traffic-light” marking on the front helpful	78.7 (72.4–84.6)	15.1 (12.9–17.2)	6.3 (4.3–8.4)
Should the state intervene more in the area of marketing to children and ban marketing of certain unhealthy foods to children (e.g., advertising sweets on children’s television, on websites, and in online games for children, on posters and products, for example through comic figures)?	53.6 (48.0–59.5)	15.1 (12.6–17.6)	31.3 (27.1–36.1)

Note: numbers are percentages of the top box values: +2 and +1 = ++/+, 0 = 0, −1 and −2 = −/−−; Confidence Interval (CI) of the lower and upper 95%, for more details of the scales please see the note of Table 5.

**Table A3:** The initial three statements of the factor ‘Perceived struggle to eat healthily’.

Factor	Statements	Factor Loading	Mean/SD
2. Perceived struggle to eat healthily (CA:0.467)			
	It’s hard for me to eat healthily.	0.875	−0.33/1.06
	Since it’s hard for me to eat healthy, the state should help.	0.606	0.34/1.11
	It is important for me to eat tasty food today, even if this is at the expense of my later health.	0.588	0.44/1.03

Notes: CA = Cronbach’s Alpha, +2 = “I totally agree” to −2 = “I do not agree at all”.
